# Lower *In vivo* Myo-Inositol in the Anterior Cingulate Cortex Correlates with Delayed Melatonin Rhythms in Young Persons with Depression

**DOI:** 10.3389/fnins.2017.00336

**Published:** 2017-06-20

**Authors:** Rébecca Robillard, Jim Lagopoulos, Daniel F. Hermens, Sharon L. Naismith, Naomi L. Rogers, Django White, Joanne S. Carpenter, Manreena Kaur, Elizabeth M. Scott, Ian B. Hickie

**Affiliations:** ^1^Institute of Mental Health ResearchOttawa, ONT, Canada; ^2^Sunshine Coast Mind and Neuroscience, Thompson Institute, University of the Sunshine CoastBirtinya, QLD, Australia; ^3^Youth Mental Health, Brain and Mind Centre, University of SydneyCamperdown, NSW, Australia; ^4^Sydney Medical School, University of SydneySydney, Australia

**Keywords:** melatonin, depression, magnetic resonance spectroscopy, myo-inositol, circadian

## Abstract

Myo-inositol, a second messenger glucose isomer and glial marker, is potentiated by melatonin. In addition to common abnormalities in melatonin regulation, depressive disorders have been associated with reduced myo-inositol in frontal structures. This study examined associations between myo-inositol in the anterior cingulate cortex and the timing of evening melatonin release. Forty young persons with unipolar depression were recruited from specialized mental health services (20.3 ± 3.8 years old). Healthy controls were recruited from the community (21.7 ± 2.6 years old). The timing of dim light melatonin onset (DLMO) was estimated using salivary melatonin sampling. Myo-inositol concentrations (MI/CrPCr ratio) in the anterior cingulate cortex were obtained using proton magnetic resonance spectroscopy. After controlling for age, sex, and CrPCr concentration the depression group had significantly lower MI/CrPCr ratios than healthy controls [*F*_(4, 75)_ = 11.4, *p* = 0.001]. In the depression group, later DLMO correlated with lower MI/CrPCr ratio (*r* = −0.48, *p* = 0.014). These findings suggest that neurochemical changes in the frontal cortex are associated with circadian disruptions in young persons with depression.

## Introduction

While disruptions in circadian rhythms have long been proposed to play a role in mood disorders, little is known about how they relate to the neurobiology of depression. At the neurochemical level, depression is often accompanied by reduced myo-inositol in the anterior cingulate cortex (ACC) and neighboring prefrontal regions, a phenomenon thought to reflect glial loss or dysfunctions (Coupland et al., 2005). Myo-inositol production and glial cell survival are both actively promoted by melatonin, a hormone also closely involved in circadian rhythms (Popova and Dubocovich, 1995; Borlongan et al., 2000). Myo-inositol production may thus be affected by the circadian disruptions commonly occurring in depression. Furthermore, since inositol trisphosphate receptors are involved in circadian entrainment (Hamada et al., 1999), depression-related changes in myo-inositol could possibly affect melatonin rhythms.

Several studies in people with depression reported reductions in melatonin secretion (Claustrat et al., 1984; Nair et al., 1984) and phase delays in the circadian rhythm of melatonin (e.g., Nair et al., 1984). Considering the typical developmental changes affecting circadian rhythms during adolescence and young adulthood (Crowley et al., 2007), youths with mood disorders may be especially sensitive to this phase delay. Accordingly, in a considerable subgroup of young people with affective disorders, dim light melatonin onset (DLMO) was found to happen after, rather than before habitual sleep onset, suggesting prominent delays in melatonin rhythms (Robillard et al., 2014). Of note, shorter phase angles between DLMO and sleep onset have also been linked to later stages of illness in youths with anxiety or depressive disorders (Naismith et al., 2012).

While melatonin is closely regulated by (and feeds back to) the biological clock in the suprachiasmatic nucleus (SCN), melatonin receptors are widely distributed across the brain, including in the frontal cortex (Mazzucchelli et al., 1996; Arendt and Skene, 2005; Uz et al., 2005). Animal studies highlighted bidirectional connections between the SCN and parts of the frontal cortex: (i) the SCN projects to cortical regions surrounding the ACC (Sylvester et al., 2002) and (ii) the anterior part of the hypothalamus, where the SCN is located, receives afferent inputs from the ACC and other medial prefrontal areas (Ongur et al., 1998). In line with the involvement of the ACC in emotional regulation (Stevens et al., 2011), these connections are thought to play a substantial role in mood variations across the 24 h cycle (Phillips et al., 2009).

Herein, we sought to investigate myo-inositol levels in the ACC and the circadian rhythm of melatonin in young persons with depression. Young people with depression were expected to have lower myo-inositol levels in the ACC as compared to healthy controls. It was further hypothesized that, in the depression group, these low levels of myo-inositol would be associated with delayed melatonin release and worse depressive symptoms.

## Materials and methods

### Participants

For the depression group, 40 help-seeking adolescents and young adults (62.5% females) receiving care for recent depressive disorders were recruited from specialized mental health services for young people (Youth Mental Health Clinic at the Brain & Mind Centre; and *headspace*, Campbelltown, Sydney, Australia). As part of the routine intake assessment of these services, diagnoses were determined by a mental health professional (i.e., clinical psychiatrists, clinical psychologists, mental health nurses, or general practitioners with a training in mental health) using criteria from the revised fourth edition of the Diagnostic and Statistical Manual of Mental Disorders (American Psychiatric Association, 2000). Each one of these participants had unipolar depression and 14 (35%) also had a comorbid anxiety disorder. Participants with comorbid bipolar or psychotic disorders were systematically excluded. Half of the participants were taking psychotropic medication. These included: antidepressants (47.5%; duloxetine, escitalopram, sertraline, paroxetine, fluoxetine), mood stabilizers/anticonvulsants (7.5%; lamortrigine, lyrica), antipsychotics (2.5%; quietiapine), benzodiazepines (5%: diazerpam, nitrazepam), and stimulants (2.5%: methylphenidate). None of the participants was taking lithium. Myo-inositol was also measured in 40 healthy controls (57.5% females) recruited from the community. Participants from the control group had no significant history of mental disorder, as self-reported over a brief telephone interview, and did not take any psychotropic medication. Across all groups, none of the participants had undertaken shift work or traveled across more than two time zones in the 60 days prior to the study. All participants provided written informed consent prior to taking part in this study. This study was carried out in accordance with the Declaration of Helsinki. All subjects gave written informed consent. Written informed consent was obtained from the parents or legal guardians of all participants who were younger than 16 years old. The protocol was approved by the Human Research Ethics Committee of the University of Sydney.

### Clinical assessment

A trained research psychologist administered the Hamilton Rating Scale for Depression (HRSD) to participants from the depression group. This 17-item scale covers a range of depressive symptoms pertaining to mood, anxiety, suicidality, appetite, sleep, somatisation, and occupational life. Total scores above 7, 16, and 23 are considered to reflect mild, moderate and severe depressive symptoms respectively. HRSD was missing for five participants (these individuals were left out of the analyses involving HRSD, yielding a sample size of 35 participants for the analysis assessing how depression severity relates to DLMO and myo-inositol).

### Magnetic resonance spectroscopy

Single voxel proton magnetic resonance spectroscopy (1H-MRS) was conducted on a 3-Tesla GE Discovery MR750 scanner using an 8-channel phased array head coil (GE Medical Systems, Milwaukee, WI) using Point RESolved Spectroscopy (PRESS) with two chemical shift-selective imaging pulses for water suppression (for detailed methodology, see Lagopoulos et al., 2013). In brief the protocol comprised a three-dimensional sagittal whole-brain scout for orientation and positioning of all subsequent scans (TR = 50 ms; TE = 4 ms; 256 matrix; no averaging, *z* = 5 mm thickness). To aid in the anatomical localisation of the MRS voxel, a T1-weighted Magnetization Prepared RApid Gradient-Echo (MPRAGE) sequence producing 196 sagittal slices (TR = 7.2 ms; TE = 2.8 ms; flip angle = 10°; matrix 256 × 256; 0.9 mm isotropic voxels) was also acquired. Finally, single voxel 1H-MRS was acquired from the anterior cingulate cortex (ACC), using an optimized PRESS acquisition (TE = 35 ms, TR = 2,000 ms, 128 averages voxel size 8 cm^3^) with two chemical shift-selective imaging pulses for water suppression. Anatomical localisation for the ACC voxel placement was based on the Talairach brain atlas and positioning was guided by the T1-weighted MPRAGE image. The ACC acquisition voxel was located anterior to the genu of the corpus callosum, oriented along the anterior-posterior commissure plane and centered on the interhemispheric fissure. MRS data was processed offline using the LCModel software package. The Cramer–Rao lower bounds remained below 20% for all participants. Creatine/phosphocreatine (CrPCr) concentrations were used as an internal standard comparator for myo-inositol, allowing the calculation of MI/CrPCr ratios. Since previous findings suggested that CrPCr concentration may differ between persons with mood disorders and healthy controls (Kato et al., 1994), we assessed group differences in CrPCr and statistically controlled for CrPCr variations in our analyses.

### Circadian assessment

The circadian assessment was conducted on average 21 days apart from MRS (SD: 34 days, range: 0–5.2 months). Following at least 7 days of sleep-wake monitoring with actigraphy (Actiwatch-64/L/2, Philips Respironics, USA), participants attended the Chronobiology and Sleep Laboratory at the Brain & Mind Centre for repeated melatonin measures under a semi-constant routine protocol. Starting 6 h before individual habitual bedtimes (based on actigraphy results), saliva samples were collected using Salivettes (*Sarstedt*, Germany) every 30-min for 8 h. Participants remained awake and were seated in dim light (<50 lux) until the end of the saliva sampling protocol. Melatonin concentrations were determined by double antibody radioimmunoassay (RKDSM2; Buhlmann Laboratories, A. G., Switzerland) with a detection threshold of 0.999 pg/mL (inter-assay coefficient of variation: 15% at 4.530 pg/mL and 12.3% at 41.114 pg/mL, intra-assay coefficient: <10% across the standard curve). DLMO was determined by linear interpolation based on the time points preceding and following the time at which melatonin concentration reached a threshold of 3.000 pg/mL, and remained above this threshold for the next three samples (Crowley et al., 2006).

### Statistical analyses

Age, time of scan, CrPCr concentration and MI/CrPCr ratios were compared between the control and depression groups with Mann-Whitney tests or independent sample *t*-tests. ANCOVAs assessing group differences were conducted on MI/CrPCr ratios while controlling for age, sex, and CrPCr concentration. Within the depression group, independent sample *t*-tests were used to compare participants taking psychotropic medication or not and those with and without comorbid anxiety disorders. Two-tailed partial correlations were conducted between DLMO timing, MI/CrPCr ratio and HRSD scores in the depression group, while controlling for age, sex, medication intake and anxiety comorbidities. All analyses were done in a pairwise manner with the Statistical Package for the Social Sciences (SPSS, version 21; IBM, USA).

## Results

### Myo-inositol in the control and depression groups

On average, the depression group was 1.7 years younger than the control group (mean age ± SD: 20.2 + 3.8 and 21.7 ± 2.6 years respectively; *p* < 0.050). There was no significant difference in the timing of scans between the depression group and the control group (*U* = 720.5, *p* = 0.437). CrPCr concentrations tended to be lower in the depression group (mean ± SD: 10.4 ± 2.3) than in the control group (mean ± SD: 11.4 ± 1.6; *U* = 601.0, *p* = 0.056). Within the depression group, no significant difference in MI/CrPCr ratios were found between participants taking and not taking psychotropic medications [*t*_(34)_ = 0.6, *p* = 0.529], nor between those with and without anxiety disorders [*t*_(38)_ = 0.4, *p* = 0.713].

**Figure 1:**
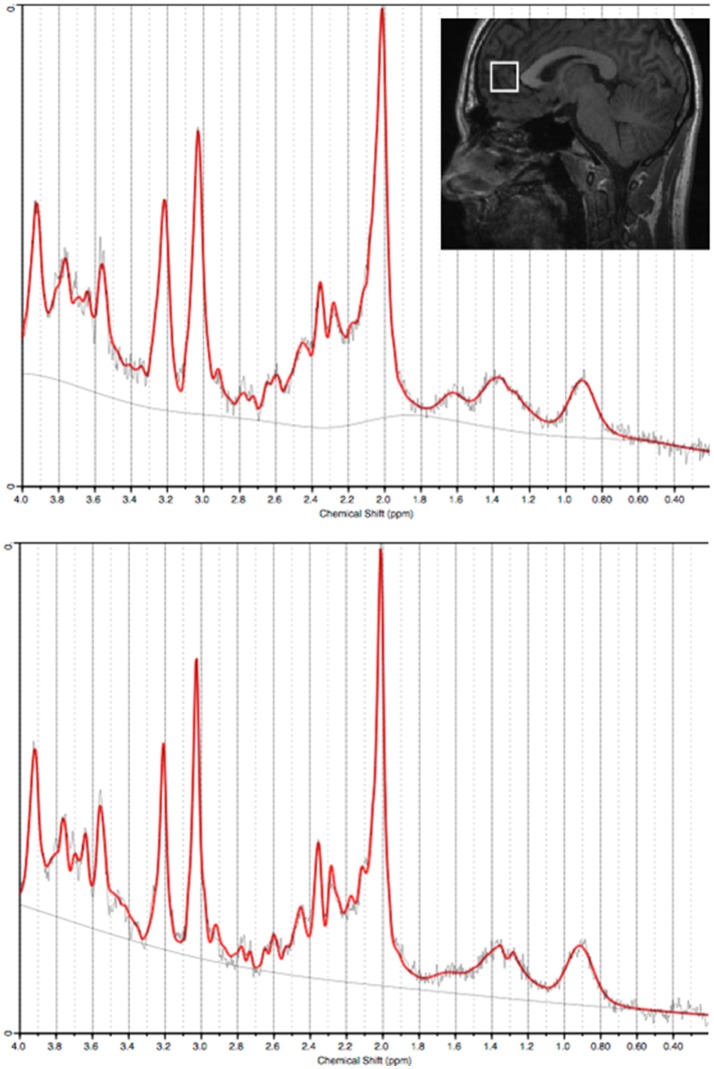
Water-suppressed spectra (from the anterior cingulate cortex) processed using LCModel from two representative subjects (**top panel:** Control; **bottom panel:** Depression). Insert: Sagittal view of a representative T1-weighted image illustrating the acquisition voxel size and placement for the anterior cingulate cortex.

Representative examples of voxel placement and water-suppressed spectra are provided in Figure 1. MI/CrPCr ratios were significantly lower in the depression group (mean ± SD: 0.84±0.10) than in the control group [mean ± SD: 0.96±0.14; *t*_(78)_ = −4.2, *p* < 0.001]. This difference remained significant after controlling for age, sex and CrPCr concentrations [*F*_(4, 75)_ = 11.4, *p* = 0.001].

### Melatonin, myo-inositol, and depressive symptoms

**Figure 2:**
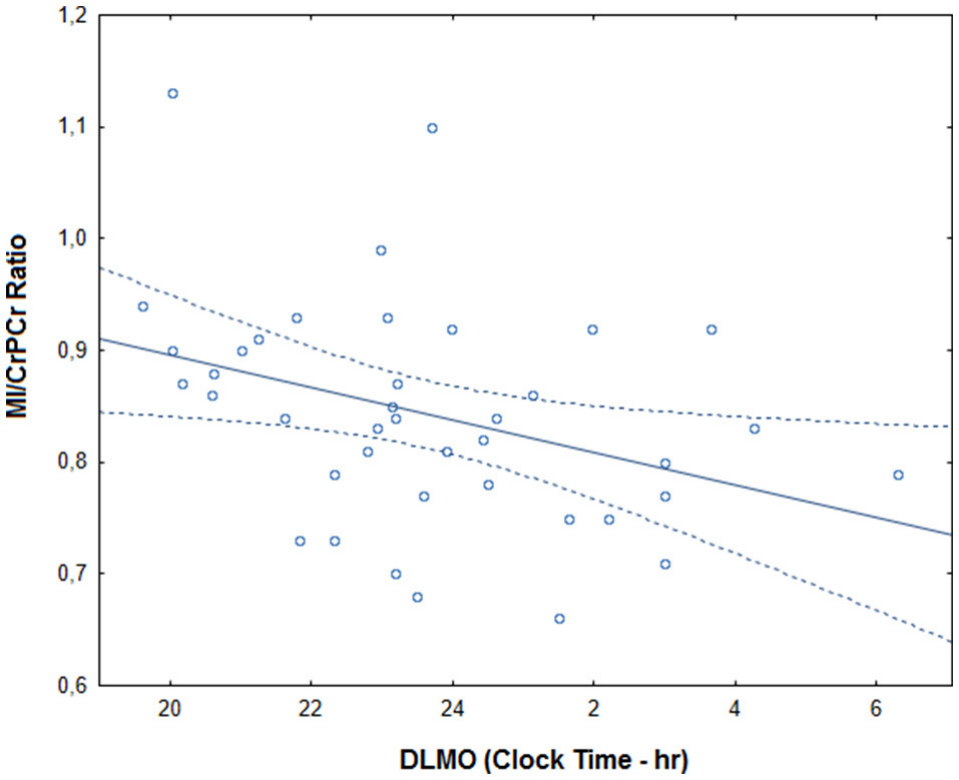
Correlation between DLMO (dim light melatonin onset) and myo-inositol in the depression group. MI/CrPCr ratio: myo-inositol relative to creatine ratio. The full line represents the fitted regression and dotted lines indicate the 95% confidence interval.

Within the depression group, there was no significant correlation between the timing of scan and myo-inositol levels (*r* = −0.23, *p* = 0.148). After controlling for age, sex, medication intake and anxiety comorbidities, later DLMO time correlated significantly with lower MI/CrPCr ratio (*r* = −0.48, *p* = 0.014; Figure 2). Total HRSD score did not correlate significantly with DLMO time (*r* = 0.26, *p* = 0.109) or MI/CrPCr ratio (*r* = −0.10, *p* = 0.534), but higher scores on the insomnia subscale correlated with later DLMO (*r* = 0.52, *p* = 0.006) and lower MI/CrPCr ratio (*r* = −0.50, *p* = 0.009). Age, sex, HRSD scores, and MI/CrPCr ratios for each individual from the depression group are provided in Supplemental Material.

## Discussion

The present findings suggest that some neurochemichal alterations in young people with depression may be associated with circadian disruptions and sleep difficulties. Specifically, decreased levels of myo-inositol in the ACC, a cortical region highly involved in emotional regulation, were found to correlate with later DLMO and more severe “insomnia” symptoms on the HRSD. This calls for further investigation of the possible interactions between melatonin, myo-inositol, and glial functions in the context of depression.

Consistent with previous studies in similar age groups (e.g., Coupland et al., 2005), we observed lower myo-inositol levels in young people with depression as compared to healthy controls. Conversely, no significant difference or increased myo-inositol levels have previously been reported in younger (e.g., Cecil et al., 2003) or older (Kumar et al., 2002) people with mood disorders. In addition, in depressed adults between 25 and 59 years old, younger age was previously found to correlate with lower myo-inositol levels in frontal regions (Frey et al., 1998). It is thus tempting to speculate that age may influence myo-inositol abnormalities linked to depression, but this remains to be further investigated.

Considering myo-inositol as a glial marker, the reduction in myo-inositol observed herein is in line with the lower glial cell density previously reported in the ACC of depressed individuals (Cotter et al., 2001; Rajkowska, 2002). As seems to be the case for myo-inositol abnormalities, there are some indications that depression-related reductions in glia are less prominent in older adults (Miguel-Hidalgo et al., 2000; Si et al., 2004; Khundakar et al., 2009, 2011). Importantly, animal studies demonstrated that experimentally induced glial ablation in the prefrontal cortex actively triggers depressive-like behaviors (Banasr and Duman, 2008), suggesting that glial loss may actively contribute to the pathogenesis of depression. The association between lower myo-inositol in the ACC and later melatonin onset may thus indicate a pathway via which circadian disruptions interact with some of the neurophysiological processes underlying depression.

Depression-related reductions in myo-inositol seem to normalize with remission (Taylor et al., 2009) and there are indications that inositol may have antidepressant properties (Levine et al., 1995; Einat et al., 1999; Chengappa et al., 2000). Animal studies suggest that exogenous melatonin actively increases myo-inositol production (Popova and Dubocovich, 1995; Bach et al., 2005). It may thus be relevant to assess whether restoring melatonin rhythms in people with depression enhances myo-inositol levels and whether this may lead to mood improvements.

The current study has several limitations. Firstly, the cross-sectional design precludes any causal inference. There was a time gap between melatonin sampling and MRS (although these measures were taken no more than 1 month apart in 87% of participants). Furthermore, psychotropic medications may possibly have affected neurometabolite and melatonin levels. Nevertheless, there were no association between medications and MI/CrPCr levels in our sample.

Overall, our findings suggest that, independently of symptom severity, neurochemical changes in the ACC in young persons with unipolar depression may be linked to delayed melatonin onset and sleep difficulties. Additional work is required to better understand the potential involvement of melatonin, myo-inositol and glial dysfunctions in the pathophysiology of depression and related sleep problems. Notably, future studies should investigate whether circadian interventions may be relevant therapeutic pathways to improve neurochemical imbalances related to depressive disorders.

## Author contributions

RR, JL and DH conducted data analyses. RR, NR, DW, JC, MK, and ES collected the data. RR, JL, NR, SN, IH contributed to the conception of the work. RR and JL drafted the work and all other authors provided critical revision of the manuscript.

### Conflict of interest statement

DH has received honoraria for educational seminars from Janssen-Cilag and Eli Lilly. ES is the (unpaid) Clinical Director of Headspace Services at the Brain and Mind Centre (BMC), the (unpaid) Coordinator of the Youth Mental Health Research Program at the BMC, and Deputy Director of St. Vincent's Private Hospital Young Adult Mental Health Unit. ES has received honoraria for educational seminars related to the clinical management of depressive disorders supported by Servier and Eli Lilly pharmaceuticals. She has participated in a national advisory board for the antidepressant compound Pristiq, manufactured by Pfizer. IH has been a Commissioner in Australia's National Mental Health Commission since 2012. He is the Co-Director, Health and Policy at the Brain and Mind Centre (BMC) University of Sydney. The BMC operates an early-intervention youth services at Camperdown under contract to headspace. IH has previously led community-based and pharmaceutical industry-supported (Wyeth, Eli Lily, Servier, Pfizer, AstraZeneca) projects focused on the identification and better management of anxiety and depression. He is a member of the Medical Advisory Panel for Medibank Private, a Board Member of Psychosis Australia Trust and a member of Veterans Mental Health Clinical Reference group. He is the Chief Scientific Advisor to, and an equity shareholder in, Innowell. Innowell has been formed by the University of Sydney and PwC to deliver the $30 m Australian Government-funded “Project Synergy”. Project Synergy is a three year program for the transformation of mental health services through the use of innovative technologies. The other authors declare that the research was conducted in the absence of any commercial or financial relationships that could be construed as a potential conflict of interest.
